# Genome-Wide Linkage Study Suggests a Susceptibility Locus for Isolated Bilateral Microtia on 4p15.32–4p16.2

**DOI:** 10.1371/journal.pone.0101152

**Published:** 2014-07-01

**Authors:** Xin Li, Jintian Hu, Jiao Zhang, Qian Jin, Duen-Mei Wang, Jun Yu, Qingguo Zhang, Yong-Biao Zhang

**Affiliations:** 1 Beijing Institute of Genomics, Chinese Academy of Sciences and Key Laboratory of Genome Science and Information, Chinese Academy of Sciences, Beijing, P. R. China; 2 Department of Cardiology, Beijing Anzhen Hospital of the Capital University of Medical Sciences, Beijing, P. R. China; 3 Department of Ear Reconstruction, Plastic Surgery Hospital, Chinese Academy of Medical Sciences, Beijing, P.R. China; Universite de Montreal, Canada

## Abstract

Microtia is a congenital deformity where the external ear is underdeveloped. Genetic investigations have identified many susceptibility genes of microtia-related syndromes. However, no causal genes were reported for isolated microtia, the main form of microtia. We conducted a genome-wide linkage analysis on a 5-generation Chinese pedigree with isolated bilateral microtia. We identified a suggestive linkage locus on 4p15.32–4p16.2 with parametric LOD score of 2.70 and nonparametric linkage score (Zmean) of 12.28 (simulated occurrence per genome scan equal to 0.46 and 0.47, respectively). Haplotype reconstruction analysis of the 4p15.32–4p16.2 region further confined the linkage signal to a 10-Mb segment located between rs12505562 and rs12649803 (9.65–30.24 cM; 5.54–15.58 Mb). Various human organ developmental genes reside in this 10-Mb susceptibility region, such as *EVC*, *EVC2*, *SLC2A9*, *NKX3-2*, and *HMX1*. The coding regions of three genes, *EVC* known for cartilage development and *NKX3-2*, *HMX1* involved in microtia, were selected for sequencing with 5 individuals from the pedigree. Of the 38 identified sequence variants, none segregates along with the disease phenotype. Other genes or DNA sequences of the 10-Mb region warrant for further investigation. In conclusion, we report a susceptibility locus of isolated microtia, and this finding will encourage future studies on the genetic basis of ear deformity.

## Introduction

Microtia includes a spectrum of congenital anomalies of the auricle, ranging from mild structural abnormalities to complete loss of the auricle (termed anotia) [1]. Microtia patients not only experience conductive hearing loss on the affected ears, but also suffer from psychosociological damage and surgical burden. The prevalence rates of microtia vary from 0.83 to 17.4 per 10,000 births worldwide, and are higher in Hispanics, Asians, Native Americans, and Andeans [2]. Most microtia studies have been performed on syndromic microtia, in contrast to the poorly studied isolated microtia [2]. One of the main reasons is the difficulty of sampling isolated microtia due to concealed deformity of external ear (less than 5% of the patients take plastic surgery). However, isolated microtia accounts for approximately 65% of all microtia cases [3], [4], and study of the isolated form of microtia may directly reveal the cause of this disease.

Microtia has heterogeneous natures in both etiology and pathology. Strong evidence showed the involvement of the genetic and environmental factors, as well as their combined effects in the disease [2]. Epidemiological studies of microtia indicated various environmental risk factors, such as lower birth weight, higher maternal age or parity, and maternal medication usage [4], [5], [6], [7]. Animal model and human genetic studies have revealed several genomic regions or genes potentially associated with microtia, such as trisomy 13 and 18, 6p24, *HOX* gene family, *SIX* gene family, *EYA*, *TBX1*, *IRF6*, *CHUK*, and *GSC* [8], [9], [10], [11], [12], [13], [14], [15], [16], [17], [18]. Although many genetic loci were reported, only two causal mutations, c.558C>A and c.703C>T, on the *HOXA2* gene were identified in syndromic microtia families [18], [19]. So far, almost all of microtia-related genes are identified from syndromes or disorders with a spectrum of anomalies, and microtia is just as one of milder phenotype of them (such as oculo-auriculo-vertebral spectrum) [20]. Most of causal genes of syndromic microtia are not replicable in isolated microtia, and no susceptibility genes or loci have been reported in association with isolated microtia till now [2].

Linkage analysis is a classic and effective way for mapping Mendelian disorders. In recent years, despite the identification of many gene variants involved in diseases through genome-wide association studies, the inability of these variants to account for much of the heritability of common disorders has led to a renewed interest in linkage analysis and other family-based methods [21]. In this study, a genome-wide linkage analysis was used to identify the susceptibility loci of microtia with a 5-generation Chinese pedigree having isolated bilateral microtia.

## Materials and Methods

### Ethics Statement

Written informed consent forms were obtained from all individuals (or their legal guardians) for genetic and biological investigations. This study was reviewed and approved by the ethics committee of the Beijing Institute of Genomics, Chinese Academy of Sciences and Plastic Surgery Hospital, Chinese Academy of Medical Science.

### Bilateral microtia pedigree

**Figure 1:**
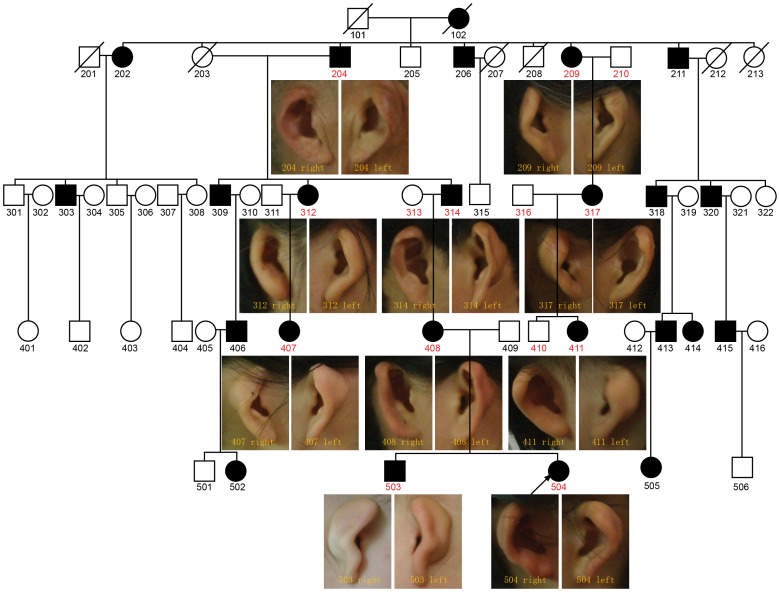
The pedigree with isolated bilateral microtia. The proband is indicated by an arrow. Individuals used in this linkage study were labeled with red symbol. The external ear of 10 genotyped patients is shown.

We recruited a five-generation pedigree with isolated bilateral microtia from Zhejiang, China (Fig. 1). All of the affected members had a normal birth without any other abnormalities. Blood samples from 14 individuals (including 10 affected and 4 unaffected individuals) of the pedigree were collected.

### Genotyping and copy number variation detection

All 14 DNA samples were extracted using DNA-extraction kits (Tiangen Biotech, Beijing, China) and genotyped on the Human Omni-Zhonghua chips (Illumina, San Diego, CA, USA) according to the manufacturer's specifications. Genotyping module of Genomestudio v3.0 (Illumina, San Diego, CA, USA) was employed to call the genotype of 900,000 SNPs. The genotypes status was estimated conditionally on the fluorescent signal and standard cluster file provided by Illumina. All DNA samples were successfully genotyped at call rate >99.7% with genotype call threshold (boundary for calling genotypes relative to its associated cluster) of 0.15. We removed SNPs (26,422) that could not be accurately clustered or were located on sex chromosomes, and obtained 873,539 SNPs for subsequent linkage analyses. We used cnvPartition 3.1.6 to detect the copy number variation (CNV) with parameters of log R ratio (logged ratio of observed probe intensity to expected intensity) and B allele frequency (the proportion of hybridized sample that carries the B allele as designated by Illumina assay). Only CNVs detected with more than 5 probes with confidence scores greater than 35 were considered.

### Linkage Analysis

To prevent false positive linkage signals due to strong linkage disequilibrium (LD) between adjacent SNP markers, we used a sliding-windows method in Plink [22] to prune our SNP set and tested as the following steps: a) calculated pairwise LD values in a window of 50 SNPs, b) removed any pair of SNPs if the pairwise LD, r^2^, was greater than 0.5, c) shifted the window 5 SNPs forward and repeated the procedure. A total of 23,139 SNPs were obtained after the sliding window test. Then, the Mendelian consistency was checked on the genotype data of each SNP with Pedcheck v1.00 [23]. The Mendelian inconsistencies rate was less than 0.09% on average, and the inconsistencies reported by Pedcheck distributed randomly across the genome and the pedigree members, indicating that the genetic relationship is as reported by the pedigree members (Table S1). Sex check was performed with both genotype and copy number information of X and Y chromosomes and revealed no sex misspecification. All markers with non-polymorphic or problematic genotypes were removed from the data set for further analyses. Multi-point linkage analyses were performed using MERLIN v.1.1.2 [24]. Parametric linkage analysis was assumed an autosomal dominant model with a risk allele frequency of 0.0001, a penetrance of 0.99 for genotypes with 1 or 2 copies of the risk allele, and a phenocopy rate of 0.001. Nonparametric linkage analysis was used to estimate the extent of allele sharing among all affected individuals with the NPL score (Zmean) and Kong and Cox's exponential LOD calculated in MERLIN[24]. The genetic positions of the markers were supplied by Illumina (HG19). The most likely haplotype was constructed using MERLIN.

### Power estimation and simulation

Power calculation was performed by SLINK (v3.00) with 1000 replicates, assuming the above autosomal-dominant model [25]. The power to detect LOD scores greater than 2 and 3 were 94.4% and 41.6%, respectively.

To determine the genome-wide significance level of the linkage signal, we performed simulation studies of 5000 replicates generated with the gene-dropping method in MERLIN. The parameters of marker allele frequency, genetic distance, pedigree structure, and missing data pattern were retained in each simulation. For individual chromosome, the proportion of simulations exhibiting equal or greater linkage scores was used to estimate the frequency of the observed linkage signal occurring by chance. Genome-wide significance levels were calculated by multiplying the chromosome-wide *P* value with a correction factor derived from the length of a simulated chromosome over the genome length in centimorgans (3,500 cM).

### Candidate gene sequencing

Candidate gene sequencing was conducted with three affected and two unaffected individuals (407, 408, 411, 313, and 316) from the pedigree. The coding regions of three candidate genes, *EVC*, *NKX3-2*, and *HMX1*, were amplified. Sequencing reactions using Applied Biosystems Big Dye Terminator chemistry were resolved on ABI Prism 3730XL DNA Analyzers (Applied Biosystems, Carlsbad, CA, USA). Phred/Phrap/Polyphred/Consed (University of Washington, Seattle, WA, USA) software was used to analyze sequence files and to call SNPs. The base-quality value threshold was set to 20 (a 99% probability of correct base calling). All polymorphic sites were manually inspected.

## Results

### Clinical Description

Our proband (6 years old) was identified as bilateral microtia. A visual malformation of external ear without other accompanying facial malformations was observed in the proband. The upper part of the affect ear was smaller than normal. Her two deformed ears measurements were same and as follows: ear length, 3.9 cm; ear width, 2.1 cm. The structure of crus helicis, cavity of concha, tragus and ear lobe were as normal, but triangular, scaphoid fossae and antihelix were absent, leaving a shell-like appearance with 130 degree of the auriculocephalic angle.

Through the proband, we identified a large microtia pedigree, which composed of 51 living individuals with 23 (12 males and 11 females) affected. All affected individuals have bilateral microtia in concha-type and the remnant external ear showed similar shell-like appearance. Only slight variation in the degree and symmetry of the affected auriculocephalic angle, auricular triangular, scaphoid fossae, and antihelix structures were observed in the affected individuals. They have no other accompanying facial malformation or hereditary disease, such as hearing loss, craniofacial deformity, and congenital heart disease. The characteristics of the affected individuals are summarized in Table 1.

**Table 1:** Ear characters of the available members of the studied microtia pedigree.

members	sex	age	ear measurements (length×width) (cm)	auriculocephalic angle	triangular	scaphoid fossae	antihelix	crus helicis	cavity of concha	tragus	ear lobe
204	male	75	5.1×2.6	100	−	−	−	+	+	+	+
209	female	56	5.0×1.9	90	−	−	−	+	+	+	+
210	male	78	6.1×3.0	60	+	+	+	+	+	+	+
312	female	49	4.5×2.3	130	−	−	−	+	+	+	+
313	female	50	5.8×2.9	55	+	+	+	+	+	+	+
314	male	51	5.1×2.0	110	−	−	−	+	+	+	+
316	male	35	5.9×3.1	45	+	+	+	+	+	+	+
317	female	32	4.6×1.9	110	−	−	−	+	+	+	+
407	female	26	5.2×2.1	125	−	−	−	+	+	+	+
408	female	27	5.2×2.2	105	−	−	−	+	+	+	+
410	male	6	5.4×2.6	50	+	+	+	+	+	+	+
411	female	4	4.1×2.2	130	−	−	−	+	+	+	+
503	male	3	4.5×2.4	130	−	−	−	+	+	+	+
504	female	6	3.9×2.1	130	−	−	−	+	+	+	+

− stands for absence/abnormal; + stands for presence/normal.

### Genome-wide linkage analysis

**Figure 2:**
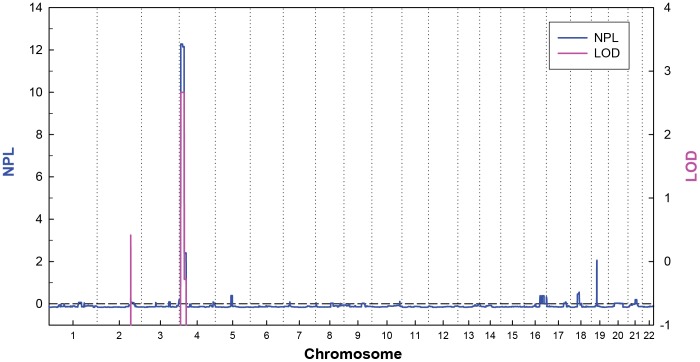
Genome-wide parametric and nonparametric linkage results of the microtia pedigree. In the parametric linkage analysis, an autosomal dominant model with a risk allele penetrance of 0.99 and a phenocopy rate of 0.001 was assumed.

We performed a genome-wide linkage analysis on this isolated microtia pedigree using a pruned set of SNPs, removal of SNPs generating strong pairwise LD (r^2^<0.5) with others. Both the parametric and nonparametric analyses indicated the highest linkage scores on chromosome 4p15.32–4p16.2 (Fig. 2), with a parametric LOD score of 2.70 (simulated occurrence per genome scan equal to 0.46) and a nonparametric linkage score of 12.28 (nominal *P*<0.00001, and simulated occurrence per genome scan equal to 0.47). According to Lander & Kruglyak [26], they proposed statistical evidence for a suggestive linkage signal would be expected to occur 0.05 to 1 time at random in a genome-wide scan. Both of our highest parametric and nonparametric linkage score, simulated 0.46 and 0.47 times per genome scan, fell within the range of suggestive linkage signal. The Kong and Cox's exponential LOD of this locus is 2.48 (simulated occurrence per genome scan equal to 0.48) at 4p15.32–4p16.2, also represents the only prominent peak of the genome-wide analysis (Table S2). Furthermore, this linkage signal is consistent in three additional analyses with different randomly selected marker sets. No other suggestive linkage signals were observed in the genome. In summary, our linkage analysis identifies a suggestive linkage signal for isolated microtia on chromosome 4p15.32–4p16.2.

### Haplotype analysis

**Figure 3:**
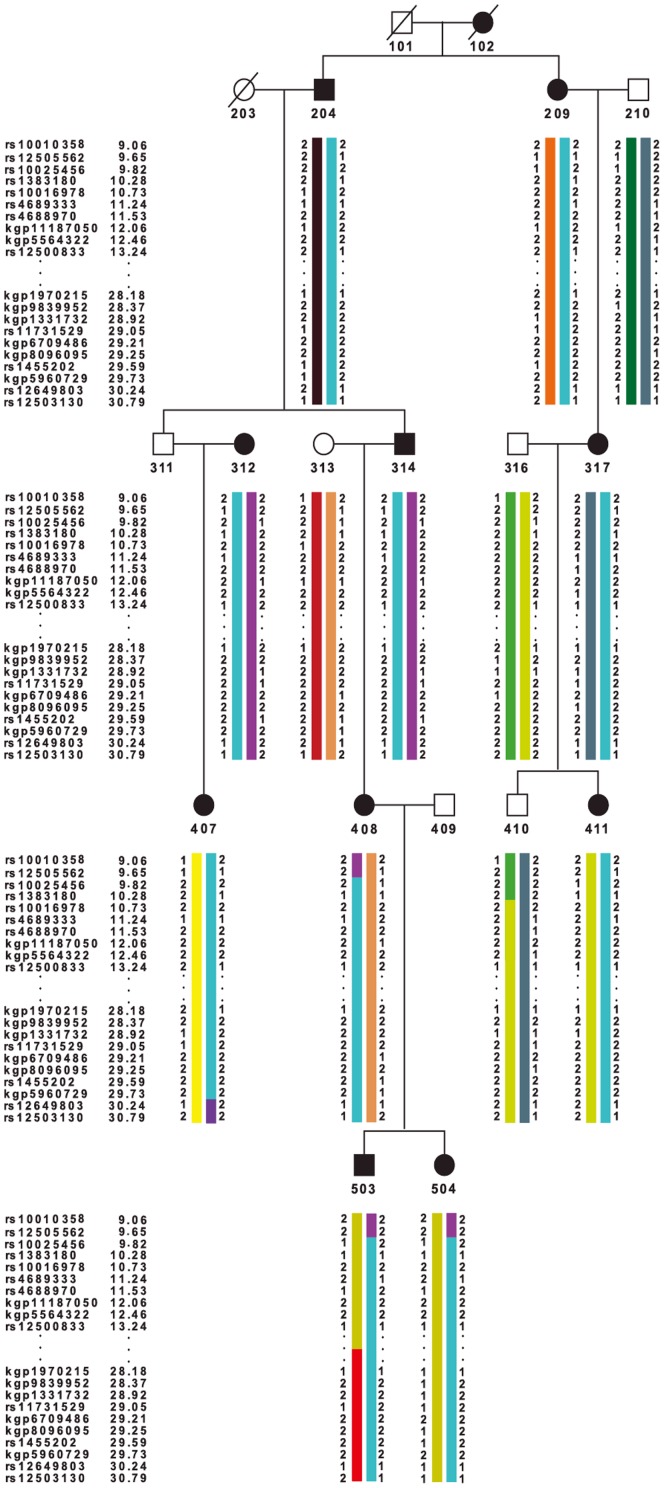
The most likely haplotypes of the microtia pedigree on chromosome 4p. The black symbols indicated the affected individuals. The disease-linked haplotype was in cyan. Marker names and their positions (cM) are listed on the left side.

To define the minimal co-segregating intervals of chromosome 4p in the affected individuals, haplotypes were constructed in the relevant genomic regions. The centromeric boundary of the interval on 4p was defined by a recombination event occurred between the SNP markers, rs12649803 and rs35933199, in the affected member, 407. The telomeric boundary of this interval corresponded to a historic recombination event occurred between rs12505562 and rs10025456 in the affected member, 408. These two events further mapped the linkage signal to a 10-Mb interval between rs12649803 and rs12505562 (Fig. 3) (9.65–30.24 cM; 5.54–15.58 Mb). All the affected individuals of the pedigree shared this disease-linked haplotype on 4p15.32–4p16.2.

### Copy number variation

We identified 19 CNVs (twelve deletions, five duplications, and two multiallelic loci) in 14 genotyped samples (Figure S1). Eight CNVs (five deletions, two duplications, and 1 multiallelic locus) were specifically carried by microtia patients, but no one CNV was carried by all patients. Within the susceptive susceptibility locus, only one duplication (copy number  = 3) that approximately ranged from chromosome 4: 8698957 to 4: 8729047 (GRCh37) was identified in patient 317.

### Genes in linkage region

There are 47 known genes in the 10-Mb linkage region. Since microtia is a developmental defect, we simply focused on genes involved in human organ development from the 10-Mb region. With the information of the function and gene ontology, we found nine organ developmental genes, *EVC*, *EVC2*, *HMX1*, *HTRA3*, *NKX3-2*, *CC2D2A*, *CRMP1*, *DRD5*, and *SLC2A9*. Of the nine genes, *EVC*, *EVC2*, *NKX3-2*, and *HMX1*, are closely related to facial development. *SLC2A9* plays roles in the development and survival of chondrocytes in cartilage matrices.

### Candidate gene sequencing

We selected coding regions of three genes from the 10-Mb region for sequencing. The *EVC* gene was selected for its roles in cartilage development, and the *NKX3-2* and *HMX1* gene was for their link to syndromic microtia [27], [28]. Thirty-two SNPs were detected in *EVC*, including 6 non-synonymous mutations and 3 synonymous mutations (Table S3). All SNPs in the coding sequence of *EVC* were present in dbSNP. In *NKX3-2*, no SNP was identified. As for *HMX1*, two SNPs recorded 5′UTR mutations was detected. All detected SNPs in three sequenced genes were not segregated with the disease status in the pedigree.

## Discussion

Isolated microtia patients (including anotia) approximately take 65% of microtia cases [3], [4]. However, few studies were performed on genetic basis of isolated microtia due to heterogeneous traits and difficult recruitment (especially for large pedigrees) of the disease. In this study, we conducted a genome-wide linkage analysis on an isolated bilateral microtia pedigree with 51 living individuals (23 patients) and revealed a suggestive linkage locus on 4p15.3–4p16.2 for human isolated microtia.

Microtia is a congenital abnormity, and genes involved in embryonic development, especially the development of first and second pharyngeal arches [2], may play a critical role of the disease. In our study, nine developmental-related genes (*EVC*, *EVC2*, *HMX1*, *HTRA3*, *NKX3-2*, *CC2D2A*, *CRMP1*, *DRD5*, and *SLC2A9*) were identified in the 10-Mb susceptibility region on 4p15.3–4p16.2. CC2D2A, DRD5, and CRMP1 are known for neural development [29], [30], [31], HTRA3 is implicated in blocking trophoblastic invasion during placental development [32]. EVC, EVC2 and SLC2A9 are critical factors in the development or survival of cartilage [33], [34], [35]. NKX3-2 and HMX1 contain a homeobox domain that involved in regulating the expression of targeted genes and directing the formation of many facial structures during early embryonic development [8], [27]. Based on the knowledge that microtia is one of the facial deformities with cartilage abnormity, we suggest detailed investigations on the *EVC*, *EVC2*, *SLC2A9*, *NKX3-2* and *HMX1* genes for this isolated microtia pedigree.

The above 5 candidate genes are associated with many facial anomalies. Defects in *EVC* or *EVC2* can cause disorders with facial abnormality, such as Ellis-van Creveld syndrome or Weyers acrodental dysostosis [36]. *NKX3-2*, expressed in lateral plate mesoderm and embryonic skeleton, plays important roles in ear morphogenesis [37], [38]. Many mutations have been identified in *NKX3-2* from disorders of oculo-auriculo-vertebral spectrum and spondylo-megaepiphyseal-metaphyseal dysplasia (both of them have facial malformation) [27], [39]. Schorderet *et al* observed *HMX1* expression in the eyes and ears of mice and human embryos. A 26-bp deletion of *HMX1* was identified as causative mutation for oculo-auricular syndrome, an autosomal-recessive disorder with malformation of eyes and ears [28]. Considering *EVC*, *NKX3-2*, and *HMX1* play key roles in facial development, we sequenced the coding regions of them with 5 family members. Although the identified mutations were not segregated well with microtia phenotype, possible causal mutations may still locate in the noncoding regions of these three genes or the other genic regions of the 4p15.3–4p16.2 (such as *EVC2* and *SLC2A9*).

Balikova et al identified a ∼750-kb amplified CNV region (approximately ranged from chromosome 4:8659910 to 4:9433782) within 4p16 was linked to syndromic microtia [40]. In our isolated microtia pedigree, patient 317 carried a duplication located in this ∼750-kb CNV region, but other patient did not. Thus, our isolated microtia pedigree was not associated with this ∼750-kb amplicon. However, we should note that omni-zhonghua might be inefficient for identifying CNVs, especially for CNVs less than 100kb, because of low SNP density [41].

In this study, we identified a novel susceptibility locus for isolated microtia. Several genes within the locus exhibit key roles in facial development and are promising for the medical genetic study of the disease. Through detecting genetic variants of these genes, we may uncover causal mutations for isolated microtia. Our results warrant further studies of the 10-Mb region in relation to external ear morphogenesis.

## Supporting Information

Figure S1 **Copy number variation map of 14 genotyped individuals.** The schematic summarizes the distribution of duplications, deletions and multi-allelic loci on each human chromosome.
(TIF)

Table S1 **The Mendelian inconsistencies rate within the pedigree.**
(DOCX)

Table S2 **The Kong and Cox's LOD score at 4p15.32–4p16.2.**
(DOCX)

Table S3 **Identified SNPs in candidate genes within susceptibility locus.**
(DOC)
