# Supplementary figures and images for: Genome-Wide Linkage Study Suggests a Susceptibility Locus for Isolated Bilateral Microtia on 4p15.32–4p16.2
Source: PLoS One. 2014 Jul 1;9(7):e101152. doi: 10.1371/journal.pone.0101152 (PMC4077761; doi:10.1371/journal.pone.0101152)

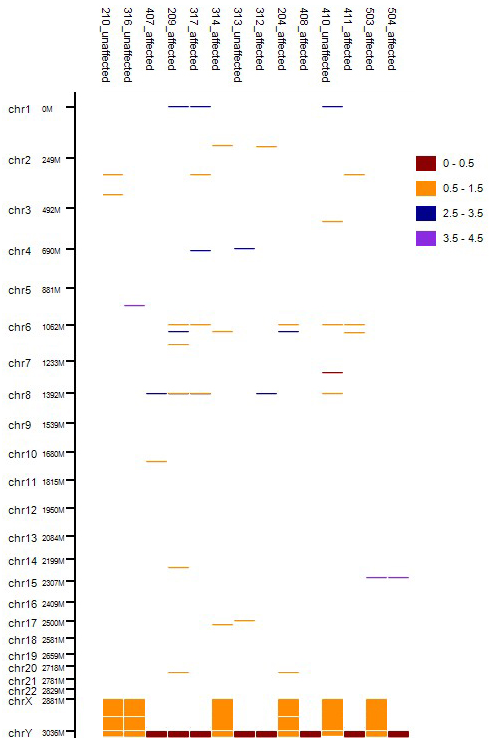

Supplement: Figure S1 — Copy number variation map of 14 genotyped individuals. The schematic summarizes the distribution of duplications, deletions and multi-allelic loci on each human chromosome. (TIF) [file pone.0101152.s001.tif]
