# Supplementary material for: Genome-Wide Linkage Study Suggests a Susceptibility Locus for Isolated Bilateral Microtia on 4p15.32–4p16.2
Source: PLoS One. 2014 Jul 1;9(7):e101152. doi: 10.1371/journal.pone.0101152 (PMC4077761; doi:10.1371/journal.pone.0101152)
Supplement: Table S1 — The Mendelian inconsistencies rate within the pedigree. (DOCX) [file pone.0101152.s002.docx]

Table S1. The Mendelian inconsistencies rate within the pedigree.

| samples | Parent-Child Error Rate (%) | Parent-Parent-Child Error Rate (%) |
| --- | --- | --- |
| 210 | 0 | 0.01323 |
| 316 | 0 | 0.36487 |
| 407 | 0.00334 | 0 |
| 209 | 0 | 0.01325 |
| 317 | 0 | 0.248 |
| 314 | 0.68819 | 0.01045 |
| 313 | 0 | 0.01047 |
| 312 | 0.00368 | 0 |
| 204 | 0.34622 | 0 |
| 408 | 0.00746 | 0.01047 |
| 410 | 0 | 0.71442 |
| 411 | 0 | 0.01571 |
| 503 | 0.00834 | 0 |
| 504 | 0.00657 | 0 |
