# Supplementary material for: Genome-Wide Linkage Study Suggests a Susceptibility Locus for Isolated Bilateral Microtia on 4p15.32–4p16.2
Source: PLoS One. 2014 Jul 1;9(7):e101152. doi: 10.1371/journal.pone.0101152 (PMC4077761; doi:10.1371/journal.pone.0101152)
Supplement: Table S2 — The Kong and Cox's LOD score at 4p15.32–4p16.2. (DOCX) [file pone.0101152.s003.docx]

Table S2. The Kong and Cox’s LOD score at 4p15.32-4p16.2

| Pos.(cM) | Zmean | *P* value | Kong and Cox's linear model | | |  | Kong and Cox's exponential model | | |
| --- | --- | --- | --- | --- | --- | --- | --- | --- | --- |
|  |  |  | Delta | LOD | *P* value |  | Delta | LOD | *P* value |
| 8.195 | 0.22 | 0.4 | 6.215 | 0.38 | 0.09 |  | 0.09 | 0.01 | 0.4 |
| 9.06 | 3.77 | 0.00008 | 6.215 | 1.39 | 0.006 |  | 3.643 | 1.95 | 0.0014 |
| 9.495 | 6.49 | 0 | 6.215 | 1.62 | 0.003 |  | 3.641 | 2.2 | 0.0007 |
| 10.124 | 11.94 | 0 | 6.215 | 1.88 | 0.002 |  | 3.641 | 2.47 | 0.0004 |
| 10.735 | 12.28 | 0 | 6.215 | 1.89 | 0.002 |  | 3.641 | 2.48 | 0.0004 |
| 12.492 | 12.28 | 0 | 6.215 | 1.89 | 0.002 |  | 3.641 | 2.48 | 0.0004 |
| 14.354 | 12.27 | 0 | 6.215 | 1.89 | 0.002 |  | 3.649 | 2.48 | 0.0004 |
| 16.259 | 12.28 | 0 | 6.215 | 1.89 | 0.002 |  | 3.65 | 2.48 | 0.0004 |
| 18.058 | 12.27 | 0 | 6.215 | 1.89 | 0.002 |  | 3.642 | 2.48 | 0.0004 |
| 21.303 | 12.16 | 0 | 6.215 | 1.88 | 0.002 |  | 0.561 | 2.38 | 0.0005 |
| 24.033 | 12.15 | 0 | 6.215 | 1.88 | 0.002 |  | 0.561 | 2.38 | 0.0005 |
| 28.065 | 12.16 | 0 | 6.215 | 1.88 | 0.002 |  | 0.56 | 2.38 | 0.0005 |
| 29.657 | 11.66 | 0 | 6.215 | 1.87 | 0.002 |  | 0.56 | 2.36 | 0.0005 |
| 29.716 | 11.2 | 0 | 6.215 | 1.85 | 0.002 |  | 0.56 | 2.34 | 0.0005 |
| 29.73 | 11.09 | 0 | 6.215 | 1.84 | 0.002 |  | 0.56 | 2.33 | 0.0005 |
| 29.861 | 10.04 | 0 | 6.215 | 1.8 | 0.002 |  | 0.56 | 2.28 | 0.0006 |
| 30.243 | 5.36 | 0 | 6.215 | 1.54 | 0.004 |  | 0.558 | 1.86 | 0.002 |
| 30.482 | 3.73 | 0.0001 | 6.215 | 1.38 | 0.006 |  | 0.555 | 1.52 | 0.004 |
| 30.607 | 2.88 | 0.002 | 6.215 | 1.28 | 0.008 |  | 0.542 | 1.09 | 0.012 |
