# Supplementary material for: Genome-Wide Linkage Study Suggests a Susceptibility Locus for Isolated Bilateral Microtia on 4p15.32–4p16.2
Source: PLoS One. 2014 Jul 1;9(7):e101152. doi: 10.1371/journal.pone.0101152 (PMC4077761; doi:10.1371/journal.pone.0101152)
Supplement: Table S3 — Identified SNPs in candidate genes within susceptibility locus. (DOC) [file pone.0101152.s004.doc]

Table S3. Identified SNPs in candidate genes within susceptibility locus.

| position | rs# | genotype | | | | | function | gene |
| --- | --- | --- | --- | --- | --- | --- | --- | --- |
|  |  | 402 | 405 | 306 | 401 | 304 |  |  |
| 4:5721021 | rs2291157 | A/C | A/A | A/A | A/A | A/C | missense | *EVC* |
| 4:5730954 | rs2276875 | G/G | G/G | G/G | G/G | A/G | intron | *EVC* |
| 4:5733068 | rs1871586 | G/G | G/G | G/T | G/T | G/G | intron | *EVC* |
| 4:5735303 | rs2291155 | C/C | C/C | C/T | C/T | C/C | intron | *EVC* |
| 4:5735316 | rs36091422 | G/G | G/G | C/G | C/G | G/G | intron | *EVC* |
| 4:5743512 | rs6414624 | C/C | C/T | T/T | C/C | C/C | missense | *EVC* |
| 4:5743580 | / | A/C | C/C | A/A | A/C | A/A | intron | *EVC* |
| 4:5746888 | / | A/T | T/T | T/T | A/T | A/T | intron | *EVC* |
| 4:5747072 | rs2286343 | T/T | T/T | T/T | T/T | T/T | intron | *EVC* |
| 4:5749784 | rs4689312 | A/A | A/A | A/A | A/A | A/G | intron | *EVC* |
| 4:5749804 | rs4689311 | A/A | A/A | A/A | A/A | A/G | intron | *EVC* |
| 4:5749814 | rs4688964 | T/T | T/T | T/T | T/T | C/T | intron | *EVC* |
| 4:5749904 | rs4688963 | C/C | C/C | C/C | C/C | C/T | synonymous | *EVC* |
| 4:5749961 | rs4688962 | C/C | C/C | C/C | C/C | C/G | synonymous | *EVC* |
| 4:5750084 | rs4688961 | T/T | T/T | T/T | T/T | A/T | intron | *EVC* |
| 4:5754396 | rs6810769 | C/C | C/C | C/C | C/C | C/G | intron | *EVC* |
| 4:5754544 | rs899691 | C/T | T/T | T/T | T/T | C/T | intron | *EVC* |
| 4:5755373 | rs7660793 | C/G | C/C | C/C | C/C | G/G | intron | *EVC* |
| 4:5755516 | rs60582583 | T/T | T/T | T/T | T/T | A/T | missense | *EVC* |
| 4:5755529 | rs116952023 | A/A | A/A | A/C | A/A | A/A | missense | *EVC* |
| 4:5755542 | rs2302075 | A/A | A/A | A/A | A/A | A/C | missense | *EVC* |
| 4:5758243 | / | G/G | G/G | G/G | T/T | G/G | intron | *EVC* |
| 4:5758274 | rs12499655 | A/A | A/A | A/A | A/A | G/G | intron | *EVC* |
| 4:5785442 | rs1383180 | A/A | A/A | A/G | A/G | A/G | missense | *EVC* |
| 4:5803633 | rs2306400 | C/C | C/C | C/T | C/T | C/C | intron | *EVC* |
| 4:5803669 | rs1031919 | T/T | T/T | C/T | C/T | T/T | intron | *EVC* |
| 4:5803830 | rs3214940 | C | C | C | delC | C | intron | *EVC* |
| 4:5803903 | rs2306399 | A/A | A/A | A/G | A/G | A/A | intron | *EVC* |
| 4:5803904 | rs1031918 | C/C | C/C | C/T | C/T | C/C | intron | *EVC* |
| 4:5806366 | rs1552057 | T/T | T/T | C/T | C/T | T/T | intron | *EVC* |
| 4:5811483 | rs28722043 | G/G | G/T | G/T | G/G | G/G | intron | *EVC* |
| 4:5812195 | rs2279250 | A/A | A/A | A/G | A/A | A/G | intron | *EVC* |
| 4:8873450 | rs4074895 | C/G | C/G | C/C | G/G | C/G | 5’UTR | *HMX1* |
| 4:8873470 | rs4074894 | A/C | A/C | A/A | C/C | A/C | 5’UTR | *HMX1* |
